# 6-Meth­oxy-1-(4-meth­oxy­phen­yl)-1,2,3,4-tetra­hydro-9*H*-β-carbolin-2-ium acetate

**DOI:** 10.1107/S1600536812016753

**Published:** 2012-04-21

**Authors:** Teik Beng Goh, Mohd Nizam Mordi, Sharif Mahsufi Mansor, Mohd Mustaqim Rosli, Hoong-Kun Fun

**Affiliations:** a Centre for Drug Research, Universiti Sains Malaysia, 11800 USM, Penang, Malaysia; b X-ray Crystallography Unit, School of Physics, Universiti Sains Malaysia, 11800 USM, Penang, Malaysia

## Abstract

In the title compound, C_19_H_21_N_2_O_2_^+^·C_2_H_3_O_2_^−^, the 1*H*-indole ring system is essentially planar [maximum deviation = 0.0257 (14) Å] and forms a dihedral angle of 87.92 (7) Å with the benzene ring attached to the tetra­hydro­pyridinium fragment. The tetra­hydro­pyridinium ring adopts a half-chair conformation. In the crystal, cations and anions are linked by inter­ionic N—H⋯O, C—H⋯O and C—H⋯N hydrogen bonds into chains along the *a* axis.

## Related literature

For the biological activity of metal complexes with 6-meth­oxy-1-methyl-4,9-dihydro-3*H*-pyrido[3,4-*b*]indole, see: Al-Allaf *et al.* (1990 ▶); Herraiz *et al.* (2003 ▶). For a related tetra­chlorido­zincate structure, see: Goh *et al.* (2012 ▶). For the stability of the temperature controller used in the data collection, see: Cosier & Glazer (1986 ▶).
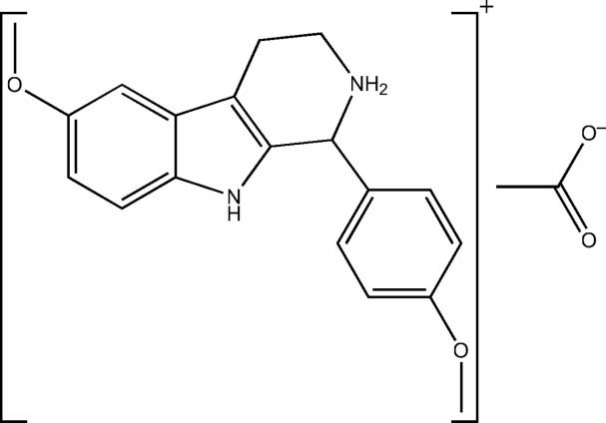


## Experimental

### 

#### Crystal data

C_19_H_21_N_2_O_2_^+^·C_2_H_3_O_2_^−^
*M* *_r_* = 368.42
Monoclinic, 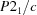

*a* = 9.1046 (4) Å
*b* = 19.8837 (8) Å
*c* = 12.0856 (5) Å
β = 123.281 (3)°
*V* = 1829.06 (15) Å^3^
*Z* = 4
Mo *K*α radiation
μ = 0.09 mm^−1^
*T* = 100 K
0.28 × 0.24 × 0.16 mm

#### Data collection

Bruker SMART APEXII CCD area-detector diffractometer
Absorption correction: multi-scan (*SADABS*; Bruker, 2009 ▶) *T*_min_ = 0.974, *T*_max_ = 0.985
21273 measured reflections
6211 independent reflections
4350 reflections with *I* > 2σ(*I*)
*R*_int_ = 0.045

#### Refinement

*R*[*F* ^2^ > 2σ(*F* ^2^)] = 0.056
*wR*(*F* ^2^) = 0.134
*S* = 1.03
6211 reflections
247 parameters
H-atom parameters constrained
Δρ_max_ = 0.42 e Å^−3^
Δρ_min_ = −0.26 e Å^−3^

### 

Data collection: *APEX2* (Bruker, 2009 ▶); cell refinement: *SAINT* (Bruker, 2009 ▶); data reduction: *SAINT*; program(s) used to solve structure: *SHELXTL* (Sheldrick, 2008 ▶); program(s) used to refine structure: *SHELXTL*; molecular graphics: *SHELXTL*; software used to prepare material for publication: *SHELXTL* and *PLATON* (Spek, 2009 ▶).

## Supplementary Material

Crystal structure: contains datablock(s) I, global. DOI: 10.1107/S1600536812016753/rz2738sup1.cif

Structure factors: contains datablock(s) I. DOI: 10.1107/S1600536812016753/rz2738Isup2.hkl

Supplementary material file. DOI: 10.1107/S1600536812016753/rz2738Isup3.cml

Additional supplementary materials:  crystallographic information; 3D view; checkCIF report

## Figures and Tables

**Table 1:** Hydrogen-bond geometry (Å, °)

*D*—H⋯*A*	*D*—H	H⋯*A*	*D*⋯*A*	*D*—H⋯*A*
N2—H1⋯O3^i^	0.93	1.86	2.7762 (15)	169
N1—H2⋯O3	0.90	1.93	2.7895 (19)	160
N2—H3⋯O4^ii^	0.97	1.72	2.6800 (18)	171
C9—H9*A*⋯O3^ii^	0.99	2.52	3.285 (2)	134
C10—H10*A*⋯N1^i^	1.00	2.55	3.4038 (19)	143
C15—H15*A*⋯O4^iii^	0.95	2.60	3.5073 (19)	160

Symmetry codes: (i) 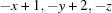; (ii) 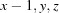; (iii) 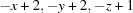.

**Fig. 1.**
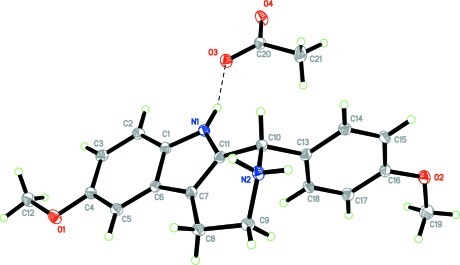
The molecular structure, showing 50% probability displacement ellipsoids. An interionic hydrogen bond is shown as a dashed line.

**Fig. 2.**
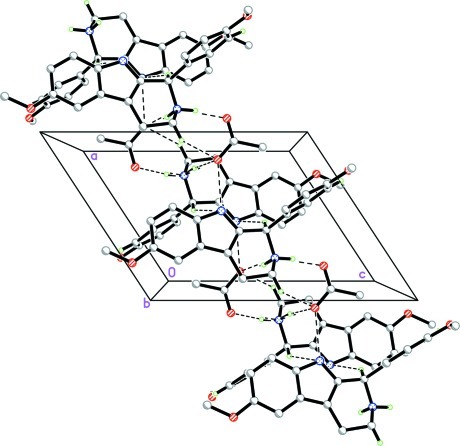
The crystal packing of the title compound. Dashed lines indicate hydrogen bonds. H atoms not involved in the hydrogen interactions have been omitted for clarity.

## supplementary crystallographic information

### Comment

The metal complexes of 6-methoxy-1-methyl-4,9-dihydro-3*H*-β-carboline and other carboline alkaloids were previously reported to have biological activity (Al-Allaf *et al.* 1990). It is now well established that these class of beta carboline alkaloids may occur under mild conditions in foods from a Pictet-Spengler condensation of indoleamines such as *L*-tryptophan and short aliphatic aldehydes (Herraiz *et al.* 2003). Our present work intend to synthesize this compound and prepare it in salt form to investigate its safety and antiproliferative efficacy in cancer cell line.

All bond lengths and angles in the title compound (Fig. 1) are within normal ranges and comparable with those observed for a related compound recently reported (Goh *et al.*, 2012). The 1*H*-indole ring (C1—C7/C11/N1) is planar with a maximum deviation of 0.0257 (14) Å for atom C11 and forms a dihedral angle of 87.92 (7)° with the C13—C18 benzene ring. The tetrahydropyridinium ring show a half-chair conformation with puckering parameters *Q* = 0.5216 (16) Å, θ = 52.70 (18)° and φ = 23.4 (2)°. In the crystal structure, cations and anions are linked by intermolecular N—H···O, C—H···O and C—H···N interactions (Table 1) into one-dimensional chains along the *a* axis (Fig. 2).

### Experimental

6-Methoxy-1-(4-methoxyphenyl)-4,9-dihydro-3*H*-β-carboline (2.50 mmol, 770 mg) was dissolved in analytical grade dichloromethane (0.60 ml). Vortex was performed to aid mixing. Glacial acetic acid (99.5%, 2.50 mmol, 145 µl) was transferred by a micropipette (50–200 µl) and was then added to the 6-methoxy-1-(4-methoxyphenyl)-4,9-dihydro-3*H*-β-carboline solution dropwise in a 20 ml glass bottle. The side of the glass bottle was scratched with a small spatula and the bottle was kept in fridge at 4° C for 60 days before yielding colourless crystals of 6-methoxy-1-(4-methoxyphenyl)-4,9-dihydro-3*H*-β-carbolinium acetate which were filtered off, washed twice with acetone and air-dried. Crystals of the title compound suitable for X-ray diffraction analysis were selected directly from the sample as prepared.

### Refinement

N-bound H atoms were located in a difference Fourier map and refined using a riding model with *U*_iso_(H) = 1.2 *U*_eq_(N). The remaining H atoms were positioned geometrically and refined using a riding model with C—H = 0.95–1.00 Å and *U*_iso_(H) = 1.2 *U*_eq_(C) or 1.5 *U*_eq_(C) for methyl H atoms. A rotating group model was applied to the methyl groups.

### Crystal data

**Table:**

C_19_H_21_N_2_O_2_^+^·C_2_H_3_O_2_^−^	*F*(000) = 784
*M**_r_* = 368.42	*D*_x_ = 1.338 Mg m^−^^3^
Monoclinic, *P*2_1_/*c*	Mo *K*α radiation, λ = 0.71073 Å
Hall symbol: -P 2ybc	Cell parameters from 5044 reflections
*a* = 9.1046 (4) Å	θ = 2.5–31.4°
*b* = 19.8837 (8) Å	µ = 0.09 mm^−^^1^
*c* = 12.0856 (5) Å	*T* = 100 K
β = 123.281 (3)°	Block, colourless
*V* = 1829.06 (15) Å^3^	0.28 × 0.24 × 0.16 mm
*Z* = 4	

### Data collection

**Table:**

Bruker SMART APEXII CCD area-detector diffractometer	6211 independent reflections
Radiation source: fine-focus sealed tube	4350 reflections with *I* > 2σ(*I*)
Graphite monochromator	*R*_int_ = 0.045
φ and ω scans	θ_max_ = 31.7°, θ_min_ = 2.1°
Absorption correction: multi-scan (*SADABS*; Bruker, 2009)	*h* = −10→13
*T*_min_ = 0.974, *T*_max_ = 0.985	*k* = −25→29
21273 measured reflections	*l* = −17→17

### Refinement

**Table:**

Refinement on *F*^2^	Primary atom site location: structure-invariant direct methods
Least-squares matrix: full	Secondary atom site location: difference Fourier map
*R*[*F*^2^ > 2σ(*F*^2^)] = 0.056	Hydrogen site location: inferred from neighbouring sites
*wR*(*F*^2^) = 0.134	H-atom parameters constrained
*S* = 1.03	*w* = 1/[σ^2^(*F*_o_^2^) + (0.0554*P*)^2^ + 0.5954*P*] where *P* = (*F*_o_^2^ + 2*F*_c_^2^)/3
6211 reflections	(Δ/σ)_max_ = 0.001
247 parameters	Δρ_max_ = 0.42 e Å^−^^3^
0 restraints	Δρ_min_ = −0.26 e Å^−^^3^

### Special details

**Table:**

Experimental. The crystal was placed in the cold stream of an Oxford Cryosystems Cobra open-flow nitrogen cryostat (Cosier & Glazer, 1986) operating at 100.0 (1) K.
Geometry. All e.s.d.'s (except the e.s.d. in the dihedral angle between two l.s. planes) are estimated using the full covariance matrix. The cell e.s.d.'s are taken into account individually in the estimation of e.s.d.'s in distances, angles and torsion angles; correlations between e.s.d.'s in cell parameters are only used when they are defined by crystal symmetry. An approximate (isotropic) treatment of cell e.s.d.'s is used for estimating e.s.d.'s involving l.s. planes.
Refinement. Refinement of *F*^2^ against ALL reflections. The weighted *R*-factor *wR* and goodness of fit *S* are based on *F*^2^, conventional *R*-factors *R* are based on *F*, with *F* set to zero for negative *F*^2^. The threshold expression of *F*^2^ > σ(*F*^2^) is used only for calculating *R*-factors(gt) *etc*. and is not relevant to the choice of reflections for refinement. *R*-factors based on *F*^2^ are statistically about twice as large as those based on *F*, and *R*- factors based on ALL data will be even larger.

### Fractional atomic coordinates and isotropic or equivalent isotropic displacement parameters (Å^2^)

**Table:**

	*x*	*y*	*z*	*U*_iso_*/*U*_eq_	
O1	0.19801 (14)	0.80635 (6)	−0.51962 (10)	0.0206 (2)	
O2	0.79721 (14)	0.79841 (5)	0.61132 (10)	0.0178 (2)	
N1	0.53529 (15)	0.91336 (6)	−0.01734 (11)	0.0134 (2)	
H2	0.6445	0.9291	0.0400	0.016*	
N2	0.21849 (15)	0.94170 (6)	0.07834 (11)	0.0140 (2)	
H1	0.1712	0.9749	0.0136	0.017*	
H3	0.2114	0.9561	0.1518	0.017*	
C1	0.47738 (18)	0.88736 (7)	−0.14119 (13)	0.0130 (3)	
C2	0.56016 (19)	0.88480 (7)	−0.20957 (14)	0.0148 (3)	
H2A	0.6767	0.9010	−0.1704	0.018*	
C3	0.46785 (19)	0.85799 (7)	−0.33659 (14)	0.0153 (3)	
H3A	0.5218	0.8559	−0.3851	0.018*	
C4	0.29553 (19)	0.83388 (7)	−0.39423 (14)	0.0147 (3)	
C5	0.21217 (19)	0.83642 (7)	−0.32661 (14)	0.0146 (3)	
H5A	0.0956	0.8201	−0.3663	0.017*	
C6	0.30332 (18)	0.86348 (7)	−0.19869 (13)	0.0121 (3)	
C7	0.25808 (18)	0.87570 (7)	−0.10371 (13)	0.0134 (3)	
C8	0.08984 (18)	0.86327 (8)	−0.11207 (14)	0.0151 (3)	
H8A	0.0532	0.8159	−0.1371	0.018*	
H8B	−0.0039	0.8926	−0.1808	0.018*	
C9	0.11639 (19)	0.87798 (7)	0.02130 (14)	0.0151 (3)	
H9A	0.0007	0.8823	0.0103	0.018*	
H9B	0.1804	0.8402	0.0828	0.018*	
C10	0.40804 (18)	0.93513 (7)	0.12006 (13)	0.0127 (3)	
H10A	0.4582	0.9815	0.1352	0.015*	
C11	0.40092 (18)	0.90627 (7)	0.00269 (13)	0.0126 (3)	
C12	0.2764 (2)	0.80697 (9)	−0.59457 (15)	0.0215 (3)	
H12A	0.1941	0.7879	−0.6825	0.032*	
H12B	0.3843	0.7801	−0.5489	0.032*	
H12C	0.3046	0.8534	−0.6038	0.032*	
C13	0.51581 (18)	0.89668 (7)	0.24838 (13)	0.0129 (3)	
C14	0.58947 (19)	0.93146 (8)	0.36854 (14)	0.0148 (3)	
H14A	0.5734	0.9787	0.3680	0.018*	
C15	0.68530 (19)	0.89756 (8)	0.48799 (14)	0.0153 (3)	
H15A	0.7354	0.9215	0.5690	0.018*	
C16	0.70801 (18)	0.82823 (7)	0.48899 (13)	0.0145 (3)	
C17	0.63921 (19)	0.79301 (8)	0.37083 (14)	0.0157 (3)	
H17A	0.6572	0.7459	0.3716	0.019*	
C18	0.54363 (18)	0.82783 (7)	0.25149 (14)	0.0146 (3)	
H18A	0.4965	0.8040	0.1707	0.018*	
C19	0.8048 (2)	0.72675 (8)	0.61618 (16)	0.0229 (3)	
H19A	0.8605	0.7118	0.7080	0.034*	
H19B	0.8737	0.7108	0.5814	0.034*	
H19C	0.6855	0.7084	0.5625	0.034*	
O3	0.88167 (13)	0.95821 (5)	0.11028 (10)	0.0169 (2)	
O4	1.16310 (14)	0.97876 (6)	0.26577 (10)	0.0201 (2)	
C20	1.00753 (19)	0.96657 (7)	0.23013 (14)	0.0147 (3)	
C21	0.9666 (2)	0.96257 (9)	0.33549 (16)	0.0247 (4)	
H21A	1.0766	0.9612	0.4231	0.037*	
H21B	0.8984	0.9218	0.3223	0.037*	
H21C	0.8984	1.0022	0.3294	0.037*	

### Atomic displacement parameters (Å^2^)

**Table:**

	*U*^11^	*U*^22^	*U*^33^	*U*^12^	*U*^13^	*U*^23^
O1	0.0193 (5)	0.0309 (6)	0.0152 (5)	−0.0070 (5)	0.0118 (5)	−0.0087 (4)
O2	0.0180 (5)	0.0186 (6)	0.0123 (5)	0.0002 (4)	0.0055 (4)	0.0019 (4)
N1	0.0116 (5)	0.0171 (6)	0.0119 (5)	−0.0026 (5)	0.0068 (5)	−0.0014 (4)
N2	0.0131 (5)	0.0170 (6)	0.0120 (5)	0.0009 (5)	0.0071 (5)	−0.0009 (4)
C1	0.0138 (6)	0.0131 (7)	0.0121 (6)	0.0008 (5)	0.0072 (5)	−0.0006 (5)
C2	0.0129 (6)	0.0167 (7)	0.0166 (7)	−0.0008 (5)	0.0091 (6)	0.0000 (5)
C3	0.0168 (7)	0.0168 (7)	0.0172 (7)	−0.0009 (6)	0.0124 (6)	−0.0006 (5)
C4	0.0160 (6)	0.0158 (7)	0.0135 (6)	−0.0011 (6)	0.0090 (6)	−0.0017 (5)
C5	0.0135 (6)	0.0167 (7)	0.0148 (6)	−0.0014 (5)	0.0085 (6)	−0.0015 (5)
C6	0.0122 (6)	0.0129 (7)	0.0124 (6)	0.0008 (5)	0.0075 (5)	0.0010 (5)
C7	0.0134 (6)	0.0157 (7)	0.0134 (6)	0.0000 (5)	0.0088 (5)	−0.0002 (5)
C8	0.0132 (6)	0.0189 (7)	0.0149 (6)	−0.0029 (5)	0.0088 (5)	−0.0029 (5)
C9	0.0130 (6)	0.0186 (7)	0.0159 (6)	−0.0019 (5)	0.0092 (6)	−0.0020 (5)
C10	0.0118 (6)	0.0144 (7)	0.0128 (6)	−0.0005 (5)	0.0073 (5)	−0.0009 (5)
C11	0.0130 (6)	0.0137 (7)	0.0126 (6)	−0.0002 (5)	0.0081 (5)	0.0004 (5)
C12	0.0241 (8)	0.0294 (9)	0.0168 (7)	−0.0058 (7)	0.0149 (7)	−0.0066 (6)
C13	0.0112 (6)	0.0163 (7)	0.0127 (6)	−0.0008 (5)	0.0077 (5)	0.0005 (5)
C14	0.0151 (6)	0.0146 (7)	0.0159 (6)	−0.0012 (5)	0.0093 (6)	−0.0016 (5)
C15	0.0149 (6)	0.0185 (7)	0.0121 (6)	−0.0037 (5)	0.0072 (5)	−0.0042 (5)
C16	0.0107 (6)	0.0202 (7)	0.0125 (6)	−0.0007 (5)	0.0063 (5)	0.0009 (5)
C17	0.0173 (7)	0.0146 (7)	0.0154 (6)	0.0006 (5)	0.0091 (6)	−0.0004 (5)
C18	0.0160 (6)	0.0155 (7)	0.0124 (6)	−0.0019 (5)	0.0078 (6)	−0.0033 (5)
C19	0.0244 (8)	0.0191 (8)	0.0199 (7)	0.0013 (6)	0.0088 (7)	0.0042 (6)
O3	0.0134 (5)	0.0211 (6)	0.0150 (5)	−0.0002 (4)	0.0071 (4)	−0.0001 (4)
O4	0.0139 (5)	0.0313 (6)	0.0166 (5)	−0.0045 (4)	0.0092 (4)	−0.0069 (4)
C20	0.0152 (6)	0.0157 (7)	0.0152 (6)	0.0003 (5)	0.0097 (6)	−0.0008 (5)
C21	0.0240 (8)	0.0365 (10)	0.0200 (8)	−0.0023 (7)	0.0163 (7)	−0.0010 (7)

### Geometric parameters (Å, º)

**Table:**

O1—C4	1.3811 (17)	C9—H9B	0.9900
O1—C12	1.4283 (17)	C10—C11	1.4982 (18)
O2—C16	1.3708 (17)	C10—C13	1.5108 (19)
O2—C19	1.4263 (19)	C10—H10A	1.0000
N1—C11	1.3784 (17)	C12—H12A	0.9800
N1—C1	1.3855 (17)	C12—H12B	0.9800
N1—H2	0.9001	C12—H12C	0.9800
N2—C9	1.4972 (19)	C13—C18	1.389 (2)
N2—C10	1.5148 (17)	C13—C14	1.4028 (19)
N2—H1	0.9296	C14—C15	1.385 (2)
N2—H3	0.9674	C14—H14A	0.9500
C1—C2	1.3918 (18)	C15—C16	1.393 (2)
C1—C6	1.4182 (19)	C15—H15A	0.9500
C2—C3	1.390 (2)	C16—C17	1.3929 (19)
C2—H2A	0.9500	C17—C18	1.393 (2)
C3—C4	1.406 (2)	C17—H17A	0.9500
C3—H3A	0.9500	C18—H18A	0.9500
C4—C5	1.3882 (18)	C19—H19A	0.9800
C5—C6	1.4001 (19)	C19—H19B	0.9800
C5—H5A	0.9500	C19—H19C	0.9800
C6—C7	1.4371 (18)	O3—C20	1.2717 (17)
C7—C11	1.3707 (19)	O4—C20	1.2573 (17)
C7—C8	1.4993 (19)	C20—C21	1.512 (2)
C8—C9	1.5213 (19)	C21—H21A	0.9800
C8—H8A	0.9900	C21—H21B	0.9800
C8—H8B	0.9900	C21—H21C	0.9800
C9—H9A	0.9900		
			
C4—O1—C12	116.63 (11)	C13—C10—N2	111.40 (11)
C16—O2—C19	117.59 (11)	C11—C10—H10A	107.7
C11—N1—C1	107.65 (11)	C13—C10—H10A	107.7
C11—N1—H2	127.8	N2—C10—H10A	107.7
C1—N1—H2	124.4	C7—C11—N1	110.93 (12)
C9—N2—C10	112.73 (11)	C7—C11—C10	125.72 (12)
C9—N2—H1	109.2	N1—C11—C10	123.06 (12)
C10—N2—H1	105.0	O1—C12—H12A	109.5
C9—N2—H3	109.6	O1—C12—H12B	109.5
C10—N2—H3	110.9	H12A—C12—H12B	109.5
H1—N2—H3	109.2	O1—C12—H12C	109.5
N1—C1—C2	130.25 (13)	H12A—C12—H12C	109.5
N1—C1—C6	108.34 (11)	H12B—C12—H12C	109.5
C2—C1—C6	121.36 (12)	C18—C13—C14	118.77 (13)
C3—C2—C1	118.27 (13)	C18—C13—C10	122.18 (12)
C3—C2—H2A	120.9	C14—C13—C10	119.04 (13)
C1—C2—H2A	120.9	C15—C14—C13	120.61 (14)
C2—C3—C4	120.81 (12)	C15—C14—H14A	119.7
C2—C3—H3A	119.6	C13—C14—H14A	119.7
C4—C3—H3A	119.6	C14—C15—C16	119.76 (13)
O1—C4—C5	115.49 (12)	C14—C15—H15A	120.1
O1—C4—C3	123.33 (12)	C16—C15—H15A	120.1
C5—C4—C3	121.18 (13)	O2—C16—C17	123.73 (13)
C4—C5—C6	118.67 (13)	O2—C16—C15	115.76 (12)
C4—C5—H5A	120.7	C17—C16—C15	120.50 (13)
C6—C5—H5A	120.7	C18—C17—C16	119.06 (14)
C5—C6—C1	119.71 (12)	C18—C17—H17A	120.5
C5—C6—C7	133.71 (13)	C16—C17—H17A	120.5
C1—C6—C7	106.56 (12)	C13—C18—C17	121.27 (13)
C11—C7—C6	106.50 (12)	C13—C18—H18A	119.4
C11—C7—C8	123.00 (12)	C17—C18—H18A	119.4
C6—C7—C8	130.41 (13)	O2—C19—H19A	109.5
C7—C8—C9	109.54 (11)	O2—C19—H19B	109.5
C7—C8—H8A	109.8	H19A—C19—H19B	109.5
C9—C8—H8A	109.8	O2—C19—H19C	109.5
C7—C8—H8B	109.8	H19A—C19—H19C	109.5
C9—C8—H8B	109.8	H19B—C19—H19C	109.5
H8A—C8—H8B	108.2	O4—C20—O3	123.85 (13)
N2—C9—C8	110.37 (11)	O4—C20—C21	118.25 (13)
N2—C9—H9A	109.6	O3—C20—C21	117.90 (13)
C8—C9—H9A	109.6	C20—C21—H21A	109.5
N2—C9—H9B	109.6	C20—C21—H21B	109.5
C8—C9—H9B	109.6	H21A—C21—H21B	109.5
H9A—C9—H9B	108.1	C20—C21—H21C	109.5
C11—C10—C13	116.38 (12)	H21A—C21—H21C	109.5
C11—C10—N2	105.57 (11)	H21B—C21—H21C	109.5
			
C11—N1—C1—C2	177.32 (15)	C6—C7—C11—N1	−0.96 (16)
C11—N1—C1—C6	−0.18 (15)	C8—C7—C11—N1	−177.99 (13)
N1—C1—C2—C3	−177.32 (14)	C6—C7—C11—C10	173.05 (13)
C6—C1—C2—C3	−0.1 (2)	C8—C7—C11—C10	−4.0 (2)
C1—C2—C3—C4	−0.2 (2)	C1—N1—C11—C7	0.73 (16)
C12—O1—C4—C5	176.19 (13)	C1—N1—C11—C10	−173.47 (13)
C12—O1—C4—C3	−3.9 (2)	C13—C10—C11—C7	108.15 (16)
C2—C3—C4—O1	−179.57 (14)	N2—C10—C11—C7	−15.97 (19)
C2—C3—C4—C5	0.4 (2)	C13—C10—C11—N1	−78.52 (17)
O1—C4—C5—C6	179.77 (13)	N2—C10—C11—N1	157.36 (13)
C3—C4—C5—C6	−0.2 (2)	C11—C10—C13—C18	−26.91 (19)
C4—C5—C6—C1	−0.1 (2)	N2—C10—C13—C18	94.16 (15)
C4—C5—C6—C7	177.79 (15)	C11—C10—C13—C14	153.50 (12)
N1—C1—C6—C5	178.05 (13)	N2—C10—C13—C14	−85.42 (15)
C2—C1—C6—C5	0.3 (2)	C18—C13—C14—C15	−1.2 (2)
N1—C1—C6—C7	−0.39 (15)	C10—C13—C14—C15	178.38 (12)
C2—C1—C6—C7	−178.16 (13)	C13—C14—C15—C16	−0.4 (2)
C5—C6—C7—C11	−177.31 (16)	C19—O2—C16—C17	−6.2 (2)
C1—C6—C7—C11	0.82 (16)	C19—O2—C16—C15	172.50 (13)
C5—C6—C7—C8	−0.6 (3)	C14—C15—C16—O2	−176.91 (12)
C1—C6—C7—C8	177.54 (14)	C14—C15—C16—C17	1.8 (2)
C11—C7—C8—C9	−9.9 (2)	O2—C16—C17—C18	177.02 (13)
C6—C7—C8—C9	173.87 (14)	C15—C16—C17—C18	−1.6 (2)
C10—N2—C9—C8	−69.07 (14)	C14—C13—C18—C17	1.4 (2)
C7—C8—C9—N2	43.56 (16)	C10—C13—C18—C17	−178.14 (13)
C9—N2—C10—C11	51.16 (14)	C16—C17—C18—C13	−0.1 (2)
C9—N2—C10—C13	−76.04 (14)		

### Hydrogen-bond geometry (Å, º)

**Table:**

*D*—H···*A*	*D*—H	H···*A*	*D*···*A*	*D*—H···*A*
N2—H1···O3^i^	0.93	1.86	2.7762 (15)	169
N1—H2···O3	0.90	1.93	2.7895 (19)	160
N2—H3···O4^ii^	0.97	1.72	2.6800 (18)	171
C9—H9*A*···O3^ii^	0.99	2.52	3.285 (2)	134
C10—H10*A*···N1^i^	1.00	2.55	3.4038 (19)	143
C15—H15*A*···O4^iii^	0.95	2.60	3.5073 (19)	160

Symmetry codes: (i) −*x*+1, −*y*+2, −*z*; (ii) *x*−1, *y*, *z*; (iii) −*x*+2, −*y*+2, −*z*+1.
